# Nanoemulsification Promotes Antibacterial Activity of Selected Essential Oils Against Multidrug‐Resistant *Staphylococcus aureus* Biofilms In Vitro

**DOI:** 10.1002/mbo3.70407

**Published:** 2026-09-09

**Authors:** Sumeena Karki, Mahnaz Ramezanpour, Clare M. Cooksley, Emma Barry, Clive A. Prestidge, Roshan Nepal, Sholeh Feizi, Dianne Correll, Peter‐John Wormald, Sarah Vreugde, Muhammed Awad

**Affiliations:** ^1^ School of Medicine, College of Health Adelaide University Adelaide South Australia Australia; ^2^ The Department of Surgery ‐ Otolaryngology‐Head and Neck Surgery, Central Adelaide Local Health Network Adeliade University and the Basil Hetzel Institute for Translational Health Research Adeliade South Australia Australia; ^3^ Centre of Pharmaceutical Innovation, School of Pharmacy and biomedical Sciences Adelaide University Adeliade South Australia Australia; ^4^ The Commonwealth Scientific and Industrial Research Organisation Hobart Tasmania Australia; ^5^ Wild Wood Oils of Australia Angaston South Australia Australia

**Keywords:** antimicrobial activity, *Artemisia vulgaris*, *Eremophila mitchellii*, nanoemulsion, *Rhododendron anthopogon*, *Staphylococcus aureus*

## Abstract

Essential oils (EOs) are unique materials with healing powers that have been extensively reported. Yet their full antimicrobial potential is still underexplored due to their complex molecular properties, high viscosity, and low solubility, which limit their wide pharmaceutical application. In this study, we report the formulation of water‐soluble nanoemulsions (NEs) of three EOs, namely, *Artemisia vulgaris*, *Rhododendron anthopogon*, and *Eremophila mitchellii*. The prepared NEs had an average particle diameter between 190 and 230 nm and remained physically stable at 4°C for up to 6 months. Most importantly, the fabricated NEs enhanced the antibacterial activity of the EOs against a multidrug‐resistant *Staphylococcus aureus* clinical isolate by eightfold, with the EO having a median minimum inhibitory concentration (MIC) of 64 mg/mL, whilst the NEs had a median value of 8 mg/mL. Moreover, the NEs reduced the biofilm viability by up to > 6 log_10_ compared with the control groups. Among the tested EOs, *E. mitchelli* showed the strongest antibacterial activity, with MIC values of 16 and < 0.125 mg/mL for the EO and NE, respectively. In addition, *E. mitchellii* NE nearly eradicated *S. aureus* biofilms at 10 mg/mL across all strains. Safety studies against human foreskin fibroblasts indicated a harmless effect of the excipients used in the fabrication process, where the viability of cells exposed to the NEs of *E. mitchellii* and *A. vulgaris* was > 70% and 90%, respectively. These findings highlight the successful fabrication of EO‐based NEs with potent antibacterial activity against multidrug‐resistant *S. aureus* clinical isolates in vitro.

## Graphical abstract

Schematic illustration of the workflow for the fabrication and biological evaluation of essential oil (EO)–based nanoemulsions (NEs). The NEs exhibited superior antibacterial activity compared with the corresponding crude EOs while maintaining high biosafety toward a skin cell line.

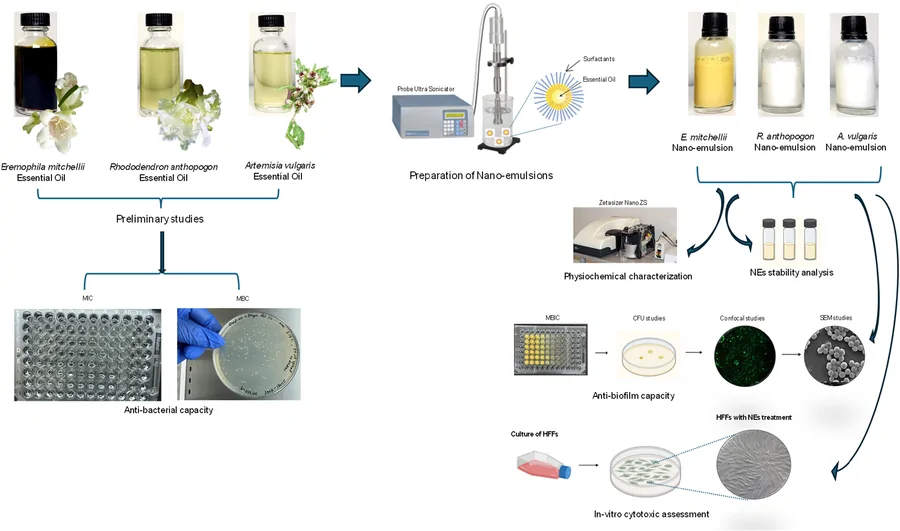

**Abbreviations**
ATCC: American Type Culture Collection
AV‐EO: *A. vulgaris* essential oil
AV‐NE: *A. vulgaris* nanoemulsion
*A. vulgaris*
: *Artemisia vulgaris*
CRS: chronic rhinosinusitis
EM‐EO: *E. mitchellii* essential oil
EM‐NE: *E. mitchellii* nanoemulsion
EO: essential oil
*E. mitchellii*
: *Eremophila mitchellii*
MAPs: medicinal and aromatic plants
NE: nanoemulsion
RA‐EO: *R. anthopogon* essential oil
RA‐NE: *R. anthopogon* nanoemulsion
*R. anthopogon*
: *Rhododendron anthopogon*
*S. aureus*
: *Staphylococcus aureus*

## 1 Introduction

Essential oils (EOs) are concentrated extracts that retain the beneficial components of their source. They are usually obtained from medicinal and aromatic plants (MAPs), which have been used as traditional medicines in various forms and for various purposes since the earliest days of mankind (Giannenas et al. 2020). About 85% of the current crude drugs used in traditional medicines are derivatives of plants, and around 60% of the world's population relies on such medications to treat or prevent diseases (Kunwar and Bussmann 2008; WHO 2022). EOs have a variety of well‐known medicinal and therapeutic properties, such as diuretic, tonic, antiseptic, and anti‐inflammatory properties (Uritu et al. 2018). Traditionally, they have been used to treat scars, infections, and pain (Muhammad et al. 2019). Recently, there has been extensive research in the food industry on the antimicrobial properties of EOs against diverse bacterial species, including pathogens that threaten human health (Mishra et al. 2020; Mitiku et al. 2026). The antimicrobial activity of EO is thought to be due to its chemical constituents, which may act synergistically against bacteria, fungi, and resistant microbial strains (Chouhan et al. 2017).

*Staphylococcus aureus* (*S. aureus*) is a common inhabitant of the human skin microbiota and a notorious bacterium commonly associated with soft‐tissue infections, including nasal and paranasal sinuses of the upper airway (Linz et al. 2023). Despite being common flora of the human nasal cavity, persistent colonization of *S. aureus*, aided by its biofilm formation, is often associated with severe chronic rhinosinusitis (CRS), a persistent inflammation of the nasal cavity and paranasal sinuses (Shaghayegh et al. 2022; Vickery et al. 2019). Further, *S. aureus* is rapidly developing resistance to commonly prescribed antibiotics like methicillin and amoxycillin, leading to hard‐to‐treat chronic infections in hospital as well as community settings (Jaradat et al. 2020). In addition to the rapid emergence of resistance, *S. aureus* can also grow in a sessile mode, in which aggregated bacterial colonies are covered by extracellular polymeric compounds known as biofilms (Otto 2008). These biofilms are commonly associated with chronic infections and can tolerate antibiotics up to 1000‐fold compared with the planktonic cells (Peng et al. 2023). Their tolerance to antibiotics usually causes infection and treatment failures (Albu 2020). Therefore, exploring the potential of nonantibiotic treatment options is a vital approach to alleviate the growing antibiotic‐resistance crisis.

EO extracted from various MAPs has long been used in traditional therapies (including Chinese medicine) and has been reported to have antimicrobial properties against both Gram‐positive and Gram‐negative bacteria (Radhakrishnan et al. 2022). Among them, *Eremophila mitchellii* (*E. mitchellii*), *Artemisia vulgaris* (*A. vulgaris*), and *Rhododendron anthopogon* (*R. anthopogon*) are some of the plants that have been used by indigenous peoples of Australia and Nepal for centuries for their medicinal values (Ekiert et al. 2020; Cock et al. 2022; Innocenti et al. 2010). EOs from these plants have been extracted from stem, leaves, and wood and are traditionally regarded as a “cough medicine” for both internal and external use to treat respiratory problems (Semple et al. 1998; Kumar et al. 2019). *E. mitchellii* has been traditionally used by indigenous Australians to treat ailments, such as cold, fever, and rheumatism, while *A. vulgaris* and *Rhododendron* spp. have long been applied in herbal medicine for their antimicrobial, anti‐inflammatory and respiratory benefits (Barnes et al. 2016; Tobyn et al. 2011; Dosoky et al. 2016). Despite multiple reports of health benefits of these EOs (Hüsnü et al. 2007), their wide application is hindered by their lipophilic nature. As such, promoting the solubility of EOs can enhance their antimicrobial activity and wider applications in clinical settings.

Recently, different approaches have been investigated to enhance the solubility of EO, including the formulation of nanoemulsions (NEs). Nanoemulsification process enhances EOs stability, solubility, and promote their interaction with biological entities via improved membrane permeability (Preeti et al. 2023). An oil‐in‐water NE is composed of oil droplets surrounded by a single layer of surfactants/cosurfactants with droplet sizes between 50 and 1000 nm (Jaiswal et al. 2015; Pavoni et al. 2020). Micelles and emulsions are different forms of colloids, where micelles of EO are smaller in size with a diameter between 2 and 100 nm, depending on the polymer composition and concentration (Perumal et al. 2022). The formulation of EO into NEs promotes the biological activity of the encapsulated oils through promoting bio‐accessibility and cellular uptake of EO active molecules (Pavoni et al. 2020; Harwansh et al. 2019). NEs are effective carriers for EOs and a promising strategy to promote their antibacterial activity, overcoming key limitations of conventional oil emulsions, such as instability, poor miscibility, and low bioavailability (Kelidari et al. 2025). These properties not only improve the solubility and stability of EO but also promote the antimicrobial efficacy of hydrophobic EO components (Garcia et al. 2022). The aim of this study is to investigate the potential of nanoemulsification in promoting the antibacterial and antibiofilm activity of the selected EOs against clinically derived *S. aureus* and its biofilms and to evaluate their in vitro safety when applied to human cell lines.

## 2 Materials and Methods

### 2.1 Patient Recruitment and Bacterial Isolates

This study was approved by the Central Adelaide Local Health Network ‐ Human Research Ethics Committee (CALHN‐HREC, Ref. 13604) and the Calvary Hospital HREC (Ref. 19‐CHREC‐E003) in Adelaide, South Australia. Following ethics approval, written consent was obtained from patients prior to the sample collection. The diagnostic criteria for CRS were obtained from the American Academy of Otolaryngology and Head and Neck Surgery and the European Position Statement (EPOS) on CRS (Fokkens et al. 2020). *S. aureus* bacteria were collected from the sinonasal cavities of CRS patients undergoing endoscopic sinus surgery. Identification of *S. aureus* (*N* = 2) was done by an independent diagnostic microbiology laboratory (The University of Adelaide, Adelaide, Australia) using matrix‐assisted laser desorption ionization‐time of flight mass spectrometry (MALDI‐TOF MS; Bruker, USA). A standard *S. aureus* ATCC25923 (American Type Culture Collection, Manassas, USA) was included as a reference strain. The clinical isolates (*S. aureus* C1033 and *S. aureus* C908) were selected based on our prior knowledge of the presence of antimicrobial resistance genes, such as *blaL* (Table S2), which provides inducible resistance to beta‐lactams antibiotics and contributes to antibiofilm production (Pence et al. 2015). The antibiotic resistance of the two clinical isolates was confirmed by testing their susceptibility to various antibiotics, including gentamycin, ciprofloxacin, clindamycin, and vancomycin, using the established broth dilution method as described by Wiegand et al. (2008) (described later), and the susceptibility was inferred according to the Clinical and Laboratory Standards Institute (CLSI) standards (M100‐Ed35) (CLSI 2025).

### 2.2 EO Sourcing

*R. anthopogon* and *A. vulgaris* oils were provided by RARA Biotech Pvt. Ltd. (Chitwan, Nepal), and *E. mitchellii* oil was provided by Wild Wood Oils (Angaston, South Australia, Australia). Working solutions of oils were prepared by diluting the oils in Mueller‐Hinton Broth (MHB) (Oxoid, Hampshire, UK. Ref. CM0405B) and vortexing for 30 s. The concentrations of EOs ranged from 0.12 to 500 mg/mL (w/v).

### 2.3 Physicochemical Characterization of Oil NEs

The EO emulsions were diluted 1:10,000 in 1.0 mM NaCl at 25°C. For standard, 1.0 mM NaCl with a refractive index of 1.33 at 25°C was used. The average particle size (*z*‐average), particle size distribution (polydispersity index, PDI), and zeta potential of EO emulsions were determined using a Zetasizer Nano ZS (Malvern, Worcestershire, UK). The experiment was conducted three times. Each measurement was performed in triplicate. All oil emulsions were sterilized using a 0.45‐µm pore syringe filter before conducting any biological experiments.

### 2.4 Preparation of Oil NEs

Surfactant Pluronic F127 (150 mg) and cosurfactant propylene glycol (PG) (1.2 g) (both Sigma‐Aldrich, St. Louis, USA) were mixed in 2.65 mL MilliQ water. The mixture was then added to 1.0 g of EO (*Rhododendron/Artemisia/Eremophila*). The final 5.0 mL solution was homogenized using a Branson Sonifier W‐450D (Hielsher, Teltow, Germany) with probe amplitude of 40%, timer on hold, duty cycle 40%, and 80 W power for 1 min. The flasks were placed in an ice bath during sonication. The sonicated samples were then stored at 4°C until further use.

### 2.5 Stability of Oil NEs

All the physicochemical parameters (*z*‐average diameter, PDI, and zeta potential) were evaluated following 1 day of fabrication, 90 and 180 days after preparation and storage at 4°C to evaluate the stability of NEs (*N* = 3) following the method using dynamic light scattering (DLS) technique as described by Wang (2024). Briefly, through DLS, the size of the particles is measured in a suspension hit with a laser, resulting in fluctuation of intensity, which is then analyzed by the nanozetsizer instrument (Stetefeld et al. 2016). The average reported values were obtained from three technical replicates.

### 2.6 Minimum Inhibitory and Bactericidal Concentration of EOs and Oil Emulsions

The antimicrobial activity of EOs and their NE was evaluated against three *S. aureus* isolates. Different concentrations of oil emulsions of *R. anthopogon*, *A. vulgaris*, and *E. mitchellii* were prepared. The minimum inhibitory concentration (MIC) was determined using the broth microdilution method with some modifications (Wiegand et al. 2008). The working concentrations of EO emulsions ranged from 1 to 128 mg/mL. A standard inoculum of *S. aureus* (1.5 × 10^8^ CFU/mL, equivalent to a 1:100 dilution of a suspension equal to a McFarland standard of 0.5) in 75 µL of MHB was added to an equal volume of each concentration of emulsions. The sterility control wells contained 150 µL of MHB. The positive solvent control was 75 µL of Pluronic F127 and PG solution in 75 µL MHB. After incubation for 18–24 h at 37°C under aerobic conditions, the lowest concentration of the emulsions that inhibited bacterial growth was designated as the MIC, following CLSI standards (M100‐Ed35) (CLSI 2025). For the minimum bactericidal concentration (MBC), 10 µL and a 1:10 dilution in MHB from each of the wells without visible turbidity was spread onto MH agar plates and incubated at 37°C for a further 24 h. Following incubation, the number of colonies obtained was compared with the number of CFU/mL in the original inoculum. The lowest concentration of emulsion that allowed < 0.1% of the original inoculum to survive was defined as the MBC. Further verification of the observed result was done by measuring the optical density at 595 nm using a plate reader (Bio‐Rad Laboratories, CA, USA). All assays were performed in triplicate.

### 2.7 Antibacterial Activity Against *S. aureus* Biofilms

*S. aureus* ATCC25923 and clinical isolates (*S. aureus* C1033 and *S. aureus* C908) were streaked in (Mueller‐Hinton agar (MHA) and incubated overnight at 37°C. Using a sterile cotton swab, a single colony of bacteria was suspended in 2 mL of saline (0.9% NaCl) to a turbidity of 2.0 ± 0.1 McFarland Units, then diluted 1:15 in MHB. For plate setup, the outer wells of a tissue culture‐treated 96‐well plate were filled with 200 µL of 1X phosphate‐buffered saline (PBS). Sterile MHB (100 µL) was used as sterility control. An additional plate was used to control the sterility of NEs at different concentrations. For biofilm establishment, 100 µL of aliquots of the diluted culture was transferred to the plate and incubated at 37°C for 24 h. Following incubation, the supernatant was discarded, and the biofilms were washed twice with 150 µL of sterile saline. Then, 100 µL of NEs at different concentrations was added to the columns and incubated for another 12–24 h. Antibiofilm activity of different NEs was tested at different MIC values. For *A. vulgaris* NE (AV‐NE), the tested concentration was 64 mg/mL (MIC for *S. aureus* ATCC25923), for *R*. *anthopogon* nanoemulsion (RA‐NE), the tested concentration was 8 mg/mL (MIC for *S. aureus* ATCC25923) and 16 mg/mL (MIC for *S. aureus* C1033). The MIC value being itself at a higher concentration for AV‐NE and RA‐NE, no further concentrations were tested for MBC. Whereas for *E. mitchellii* NE (EM‐NE), 0.12 mg/mL (MIC for *S. aureus* ATCC25934) was selected with an increment of 10X. The value was selected because our previous study by Awad et al. (2022) found that a concentration of 10 times the MIC significantly reduced *S. aureus* biofilm viability compared with lower concentrations (e.g., 2–5X MIC). As such, EM‐NE at 0.12, 1, 5, and 10 mg/mL was tested. Bacteria were used only as a positive growth control, and a separate plate with different concentrations of NEs was also incubated as a sterility control. All the plates were wrapped with aluminum foil (to prevent evaporation) and incubated overnight at 37°C on a rotating plate set at 70 rpm (3D Gyratory Mixer, Ratek Instruments, Australia). Following incubation, the supernatant was discarded, and the biofilms were washed twice with 200 µL 1X PBS without disturbing the biofilm. Then, the biofilms were extracted from each well by scraping with sterile pipette tips and serially diluted in 1X saline (0.9% NaCl), serially diluted and plated for colony‐forming unit (CFU) enumeration. Bacterial viability was assessed using three technical replicate wells, each containing three isolates. The concentration of NEs completely eardicating biofilms (i.e., giving 0 growth in the assay) was defined as the minimum biofilm eradication concentration (MBEC) observed in the assay. In cases of low readings of the number of growths, even at the highest concentration of NEs, we expressed them as effective values, defined as the final concentration observed in the reduction of biofilm formation compared with the untreated control.

### 2.8 Confocal Microscopy

A single colony of *S. aureus* C1033 was incubated in MHB at 37°C for 16–18 h. The bacterial suspension was adjusted to 1.0 ± 0.1 McFarland units, equivalent to 3 × 10^8^ CFU/mL, followed by 1:15 dilution in MHB. This bacterial suspension (200 µL) was incubated in an eight‐well slide chamber (Thermo Scientific Nunc Lab‐Tek with cover) for 24 h at 37°C on a rotating plate set at 70 rpm (3D Gyratory Mixer, Ratek Instruments, Australia) to allow biofilm formation. Following incubation, biofilms were washed twice with 1X saline to remove excess planktonic bacteria, and treatments (EO or NE) were added and incubated for 24 h at 37°C on a rotating plate. Treatments of EM‐EO and EM‐NE at concentrations 1, 5, and 10 mg/mL were prepared from a stock of 128 mg/mL diluted in MHB. The biofilms were then washed twice with 1X saline to remove the treatments and fixed with 5% glutaraldehyde (Sigma‐Aldrich, USA) for 45 min at room temperature (RT). The cells were again washed with 1X saline and stained with 200 µL equimolar solutions of Syto‐9 green (live stain) and propidium iodide (dead stain) (Invitrogen Molecular Probes, Mulgrave, Victoria, Australia) for 15 min in the dark, followed by washing with 1X saline to remove excess stains. The chambers were removed from the glass slide, and fluorescence enhancer solution was added, and then covered with a glass coverslip. The biofilms were imaged by confocal microscope (LSM900, Zeiss, Ober, Germany) at specified excitation emission wavelengths.

### 2.9 Scanning Electron Microscopy (SEM)

SEM was conducted to examine the action of EM‐EO and EM‐NE on *S. aureus* C1033 cell membranes. An overnight culture of *S. aureus* C1033 was adjusted to 1.0 ± 0.1 McFarland units, followed by 1:15 dilution in MHB. Aliquots (400 μL) of this bacterial suspension were transferred to individual wells in a 12‐well plate containing sterile coverslips (10 mm × 10 mm) and incubated at 37°C for 48 h on a rotating plate set to 70 rpm (3D Gyratory Mixer, Ratek Instruments, Australia) to allow biofilm growth. After incubation, the wells were washed twice with sterile PBS (0.1 M, pH 7.0). Treatments of each EM‐EO and EM‐NE were prepared at concentrations of 0.1, 1, 5, 10, and 128 mg/mL diluted in MHB. The 400 μL/well treatments were added to the wells and incubated at 37°C for 12 h on a rotating plate set to 70 rpm (3D Gyratory Mixer, Ratek Instruments, Australia). For the negative control, fresh MHB was added to the well. All wells were aspirated and washed twice with 500 μL PBS. The cells were then fixed with a mixture of 4% paraformaldehyde, 1.25% glutaraldehyde, and 4% sucrose on 0.05 M phosphate buffer (pH 7.2) and placed at 4°C for 12 h. After washing the cells twice with washing buffer (PBS with 4% sucrose) at interval of 5 min, the cells were treated with 2% osmium tetroxide for 1 h and dehydrated through a graded series of ethanol solutions (50%, 70%, 95%, and absolute) and then placed in 50% hexamethyldisilazane (HMDS) in absolute ethanol for 10 min followed by two changes of 100% HMDS for 10 min each. Finally, HMDS was removed from the samples, which were then allowed to dry at RT. Coverslips were mounted on aluminum stubs and coated with 5 nm (diameter) platinum nanoparticles using a 208 High Resolution Cressington sputter coater. Imaging was performed using the Quanta FEG 450 Environmental SEM at Adelaide Microscopy, Adelaide University.

### 2.10 Cytotoxicity Assay

Human foreskin fibroblasts (HFFs) were obtained from the Basil Hetzel Institute (BHI), Australia, collection with compliance with the protocol set by BHI at Adelaide University. The HFF cells were cultured and treated according to a standard in‐house‐optimized protocol by Awad et al. (2022). Briefly, the HFF cells were seeded (20,000 cells/mL) in 96‐well plates and incubated at 37°C for 24 h with 5% CO_2_. After 24 h, the cell culture medium was removed, and the cells were treated with 100 µL of AV‐NE (64 mg/mL), RA‐NE (8 mg/mL), and EM‐NE (0.12 and 1 mg/mL) solubilized in Dulbecco's modified Eagle's medium (DMEM) (STEMCELL Technologies, Tullamarine, Vic, Australia). The treatments were kept for 5 to 60 min. The treatments were removed at subsequent time points, and 5 mg/mL of 3‐(4,5‐dimethlythiazil‐2‐yl)–2,5‐diphenyltetrazolium bromide (MTT) was added to the subsequent wells, solubilized in DMEM media, and incubated for another 4 h in an incubator. Following incubation, the supernatant was removed, and cells were lysed with 100 µL dimethyl sulfoxide by incubating at RT on a rotating plate set at 70 rpm (3D Gyratory Mixer, Ratek Instruments, Australia) for 30 min. Subsequent analysis was performed with absorbance measured at 570 nm using a microplate reader (Bio‐Rad, California). Positive control contained fibroblast cells with DMEM with 10% fetal bovine serum (FBS), and negative control contained 1% Triton X‐100 in DMEM media. The percentage of cell viability was calculated by following the formula:

$$\text{Percentage cell viability}=\frac{\left(\text{Mean absorbance of sample}-\text{Mean absorbance of blank}\right)}{\left(\text{Mean absorbance of control}-\text{Mean absorbance of blank}\right)}\times 100.$$

## 3 Results

### 3.1 Physicochemical Characterization of NEs

**Figure 1:**
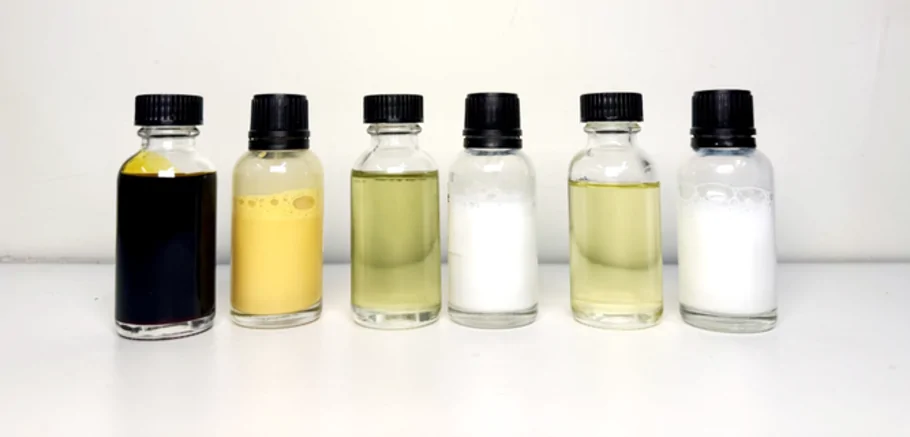
Physical appearance of essential oil (EO) and its nanoemulsion (NE) formulations. From left to right: AV‐EO, *Artemisia vulgaris* essential oil; AV‐NE, *A. vulgaris* nanoemulsion; EM‐EO, *Eremophila mitchellii* essential oil; EM‐NE, *E. mitchellii* nanoemulsion; RA‐EO, *Rhododendron anthopogon* essential oil; RA‐NE, *R. anthopogon* nanoemulsion.

All prepared EO NEs maintained their emulsified state, exhibiting characteristics of a water‐based dispersion. The visual appearance of emulsified EO of *A. vulgaris* and *R. anthopogon* was milky white, whereas that of *E. mitchellii* was cloudy yellow (Figure 1). All EO NEs showed an average particle size less than 240 nm, a negative zeta potential, and a PDI value lower than 0.3. Specifically, the *z*‐average diameter of *A. vulgaris, E. mitchellii*, and *R. anthopogon* EO emulsions was 217.2 ± 9.53, 230.4 ± 6.44, and 192.9 ± 11.11 nm, respectively, which indicates the successful formulation of nano‐sized emulsions (Table 1). The PDI values were 0.196 ± 0.02, 0.133 ± 0.01, and 0.175 ± 0.10 while zeta‐potential values were −0.386, −18.366, and −2.883 mV for *A. vulgaris, E. mitchellii*, and *R. anthopogon* EO emulsions, respectively (Table 1). A lower PDI indicates higher uniformity (particle size), and a high *z*‐potential (> ±10 mV) indicates stable colloidal dispersion of the EO NEs. Amongst all three tested EO NEs, *E. mitchellii* NEs showed the highest droplet size (230.4 ± 6.44), negative *z*‐potential (−18.366), and the smallest PDI (0.133 ± 0.01).

**Table 1:** Physiochemical characteristics of freshly prepared nanoemulsions prepared from *Artemisia vulgaris*, *Eremophila mitchellii*, and *Rhododendron anthopogon* essential oils.

Plant	Freshly prepared oil nanoemulsions		*z*‐Potential (mV)
	*z*‐Average (nm)	Polydispersity index	
*A. vulgaris*	217.2 ± 9.53	0.196 ± 0.02	−0.386
*E. mitchellii*	230.4 ± 6.44	0.133 ± 0.01	−18.366
*R. anthopogon*	192.9 ± 11.11	0.175 ± 0.10	−2.883

*Note:* mV = millivolt; nm = nanometer; *z*‐average = intensity‐weighted mean hydrodynamic size of a nanoparticle; *z*‐potential or zeta‐potential = surface electrical charge of nanoparticle.

Further, to evaluate the physical stability of all three EO NEs over time, their physicochemical characteristics were measured after storage for 3 and 6 months (Table 2). All three EO‐NEs remained physically stable for up to 6 months, presenting minimum change to their physical characteristics (average size less than 340 nm, negative zeta potential, and PDI less than 0.3 compared with fresh NEs, Table 1), indicating the stability of the formulations and uniformity of their particle size over time.

**Table 2:** Physiochemical characteristics of nanoemulsions prepared from *Artemisia vulgaris*, *Eremophila mitchellii*, and *Rhododendron anthopogon* essential oils after 3 and 6 months.

Plant	Three‐months nanoemulsion		Six‐months nanoemulsion	
	*z*‐Average (nm)	Polydispersity index	*z*‐Average (nm)	Polydispersity index
*A. vulgaris*	273.9 ± 3.80	0.144 ± 0.09	242.6 ± 2.57	0.213 ± 0.01
*E. mitchellii*	337.6 ± 28.92	0.350 ± 0.03	330.7 ± 31.0	0.342 ± 0.02
*R. anthopogon*	307.4 ± 16.88	0.358 ± 0.009	218.7 ± 9.47	0.338 ± 0.02

*Note:* mV = millivolt; nm = nanometer; *z*‐average = intensity‐weighted mean hydrodynamic size of a nanoparticle.

### 3.2 Antibacterial Activity of EOs and Their NEs

#### 3.2.1 Planktonic Antibacterial Activity

Antibiotic susceptibility test (AST) indicated that the clinical isolates *S. aureus* C1033 was resistant to clindamycin (MIC = 1 μg/mL) and vancomycin (MIC = 4 μg/mL) and *S. aureus* C908 was resistant to gentamycin (MIC = 8 μg/mL), ciprofloxacin (MIC = 2 μg/mL), clindamycin (MIC = 2 μg/mL), and vancomycin (MIC = 4 μg/mL) (Table S3) and thus were designated as multidrug‐resistant. We evaluated the antibacterial activity of the EO and its freshly formulated NE against these two *S. aureus* clinical isolates and a standard *S. aureus* ATCC25923. The solubility effects of EOs and NEs were different from MHB. EOs had less solubility with increased separation rate at higher concentration of EO, whereas both solubility and stability of NEs persisted after dilution with MHB, which enhanced their efficacy against S*. aureus*.

The three crude EOs showed variable activity against the tested bacteria with MIC values of 512 mg/mL for *A. vulgaris*, 16–64 mg/mL for *E. mitchellii*, and 32–128 mg/mL for *R. anthopogon* EOs. On the other hand, MIC values for NEs were significantly lower in comparison to their respective EOs, with values ranging between 64 and 128 mg/mL for *A. vulgaris* NE (AV‐NE), < 0.125–1 mg/mL for *E. mitchellii* NE (EM‐NE), and 4–8 mg/mL for *R. anthopogon* NE (RA‐NE). Among the three EOs and their NEs tested, *E. mitchellii* NE demonstrated the strongest antibacterial activity with MIC values at 1 mg/mL or lower (Table 3). Furthermore, we assessed the stability of NEs antibacterial performance, and the MIC values were examined after 6 months of storage. There was a minimal change in MIC values for AV‐NE and EM‐NE but a significant increase in MIC value of RA‐NE against *S. aureus* clinical isolate C908, that is, MIC increased from 8 to 64 mg/mL (Table S1).

**Table 3:** The antimicrobial activity of essential oil (EO) and nanoemulsion (NE) of *Artemisia vulgaris*, *Eremophila mitchellii*, and *Rhododendron anthopogon* against *Staphylococcus aureus* ATCC25923 and clinical isolates C1033 and C908.

Plants	Bacterial isolate	Minimum inhibitory concentration	
		EO (mg/mL)	NE (mg/mL)
*A. vulgaris*	*S. aureus* ATCC25923	512	64
	*S. aureus* C1033	512	64
	*S. aureus* C908	> 512	128
*E. mitchellii*	*S. aureus* ATCC25923	16	< 0.125
	*S. aureus* C1033	32	1
	*S. aureus* C908	64	1
*R. anthopogon*	*S. aureus* ATCC25923	32	4
	*S. aureus* C1033	64	8
	*S. aureus* C908	128	8

*Note:* Working concentration of EO = 1 g/mL in tryptone soy broth and NE = 200 mg/mL of nanoemulsified EO.

#### 3.2.2 Antibiofilm Activity

**Figure 2:**
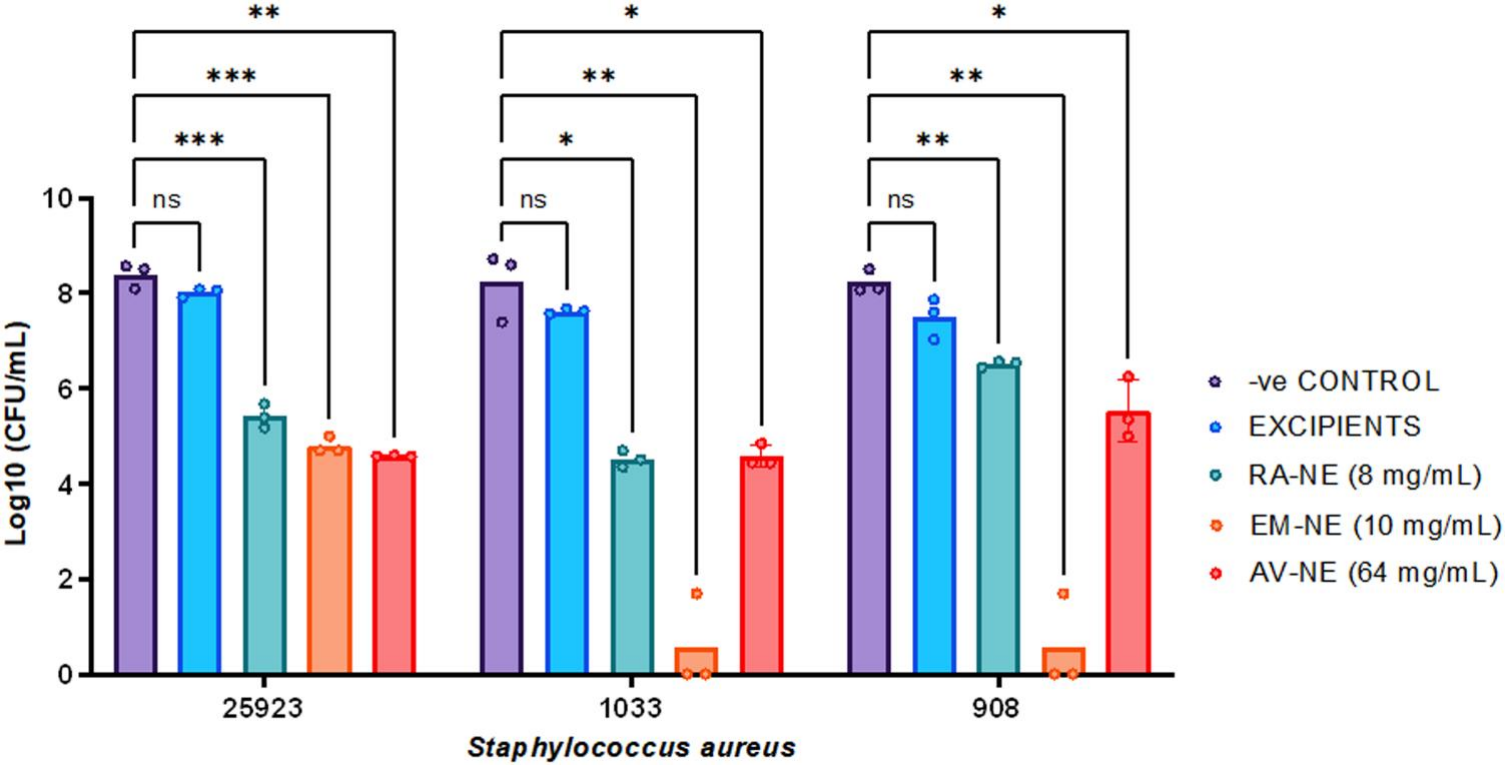
Antibiofilm activity of nanoemulsions (NE) against *Staphylococcus aureus*. Activity of *Rhododendron anthopogon* nanoemulsion (RA‐NE) at 8 mg/mL, *Eremophila mitchellii* nanoemulsion (EM‐NE) at 10 mg/mL, and *Artemisia vulgaris* nanoemulsion (AV‐NE) at 64 mg/mL against standard *S. aureus* ATCC25923 and clinical isolates C1033 and C908. Data presented as mean ± SD of three biological replicates. ns, nonsignificant; *significant reduction in viability; **p = 0.0012; ***p < 0.0001 (two‐way ANOVA test followed by multiple comparison Dunnett's test). ANOVA, analysis of variance.

The antibiofilm activity of the NEs was assessed against established *S. aureus* biofilms. As a previous study by Awad et al. (2022) indicated that higher concentrations (e.g., 10X MIC) were more effective against established biofilms than lower concentrations (e.g., 2–5X MIC), we used the respective values for the antibiofilm assay. Since the MIC values of *A. vulgaris* NE (AV‐NE) and *R*. *anthopogon* NE (RA‐NE) against the *S. aureus* ATCC25923 strain were relatively high, at 64 and 8 mg/mL, respectively, they were used to assess antibiofilm activity. Similarly, *E. mitchellii* NE (EM‐NE) was tested at the MIC value (0.12 mg/mL), with increments of 10X (1 and 10 mg/mL). Our study determined a direct correlation between EO concentration in the formulation and antibiofilm activity when EM‐NE were exposed to increasing concentrations of 0.12, 1, 5, and 10 mg/mL. It showed varying degrees of reduction in bacterial viability (Figures 2 and S1). At 10 mg/mL, EM‐NE showed the highest antibiofilm activity with a steep drop in the CFU counting, recording 3.5, 7.6, and 8.3 log_10_ reduction for *S. aureus* ATCC25923, *S. aureus* C1033, and *S. aureus* C908 isolates, respectively (*p* < 0.0001) (Figure 2).

Similarly, AV‐NE significantly reduced the viability of biofilms, recording a 3.8 log_10_ reduction in viability of *S. aureus* ATCC25923 (Figure 2) (*p* < 0.0001), a 3.6 and 3.1 log_10_ reduction for *S. aureus* C1033 and *S. aureus* C908 strains (*p* < 0.01) compared with their respective positive control (Figure 2). RA‐NE reduced the biofilm viability for all three *S. aureus* isolates by up to 3.2 log_10_ (*p* < 0.0001) (Figures 2 and S1). Unlike NEs, the excipients (Pluronic F127 and propylene glycol) did not show any antibiofilm activity even at the highest concentration (24% v/v, diluted in MHB used during the formulation), that is, it was ineffective against biofilms compared with positive controls (*S. aureus* ATCC25923, *S. aureus* C1033, and *S. aureus* C908) when applied for 24 h (Figure 2).

#### 3.2.3 *S. aureus* Biofilms Morphology Using Confocal and SEM

**Figure 3:**
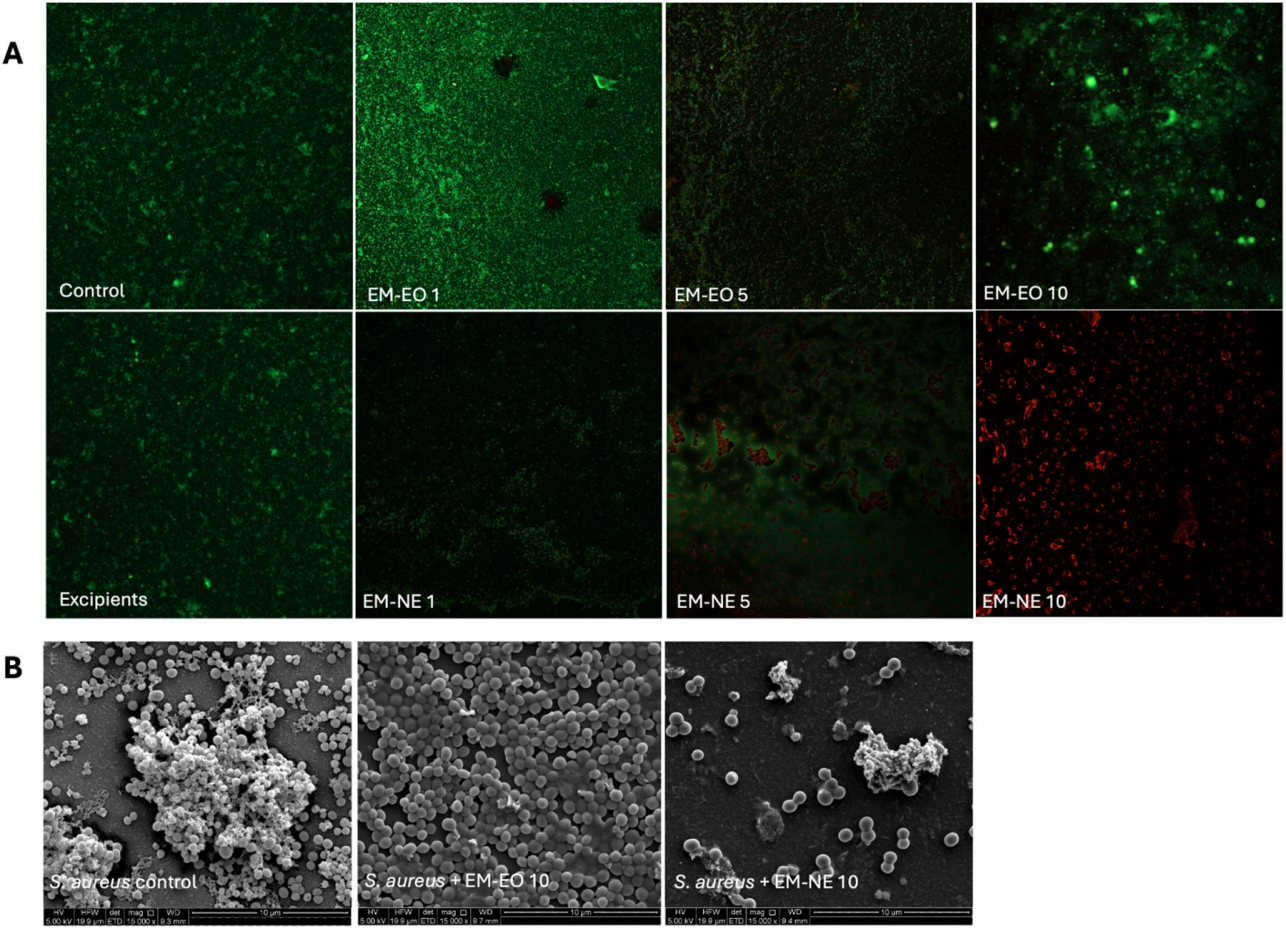
Confocal and scanning electron micrographs of *Staphylococcus aureus* and its biofilm. (A) Confocal images of *S. aureus* C1033 biofilms stained with live stain Syto9 (green) and dead stain propidium iodide (red), following 24 h exposure to different concentrations (1, 5, and 10 mg/mL) of *Eremophila mitchellii* essential oil (EM‐EO) and *E. mitchellii* nanoemulsion (EM‐NE). (B) Scanning electron microscope images showing the morphology of *S. aureus* C1033 biofilms with different treatments at ×15 magnification; control biofilms (*S. aureus* C1033 with no treatment), biofilms after treatment with unformulated EM‐EO at 10 mg/mL for 12 h), and biofilms after treatment with EM‐NE containing 10 mg/mL. The images depict the disrupted biofilm matrix and distorted *S. aureus* cells after the treatment.

In addition to the CFU estimation, the antibiofilm activity of the NEs was further investigated using a live/dead staining assay, and the biofilms were imaged using confocal microscopy. *E. mitchellii* EO and its NE were investigated as the model due to the highest antibiofilm activity. In agreement with CFU data, EM‐NE showed improved reduction in viability compared with crude EO, with a dose‐dependent antibiofilm activity of the EO. At 1 mg/mL of EO, there was no significant difference in SYTO‐9 (live stain) compared with the control. However, at 5 mg/mL EM‐NE, a prominent antibiofilm activity was observed, whereas at 10 mg/mL EM‐NE, biofilm was completely eradicated with no live cells detected in the biofilm (Figure 3A).

SEM was utilized to capture the morphology of *S. aureus* C1033 biofilms following treatment with unformulated *E. mitchellii* EO and NEs. The morphology of the control (untreated) cells had a typical round structure (Figure 3B), while treatment with EM‐EO at 10 mg/mL caused a significant reduction in cell number and morphological changes (Figure 3B). Further, we observed enhanced biofilm matrix disruption in the cells treated with EM‐NE at the same concentration with considerable distorted cells (Figure 3B). Cell constituents seemed to be less with a lower concentration of EM‐EO (< 5 mg/mL), and as the concentration of EOs was increased, there were no inhibitory effects on *S. aureus* (Figure S2). The case seemed to be reversed with EM‐NE, where biofilms were disrupted, showing clearly damaged cell surfaces and cell walls after treatment with EM‐NE at different concentrations (0.1, 1, and 5 mg/mL). The bacterial morphology was distorted in correlation with the increased concentration of NE (Figures S2 and 3).

### 3.3 Cytotoxicity Assay

**Figure 4:**
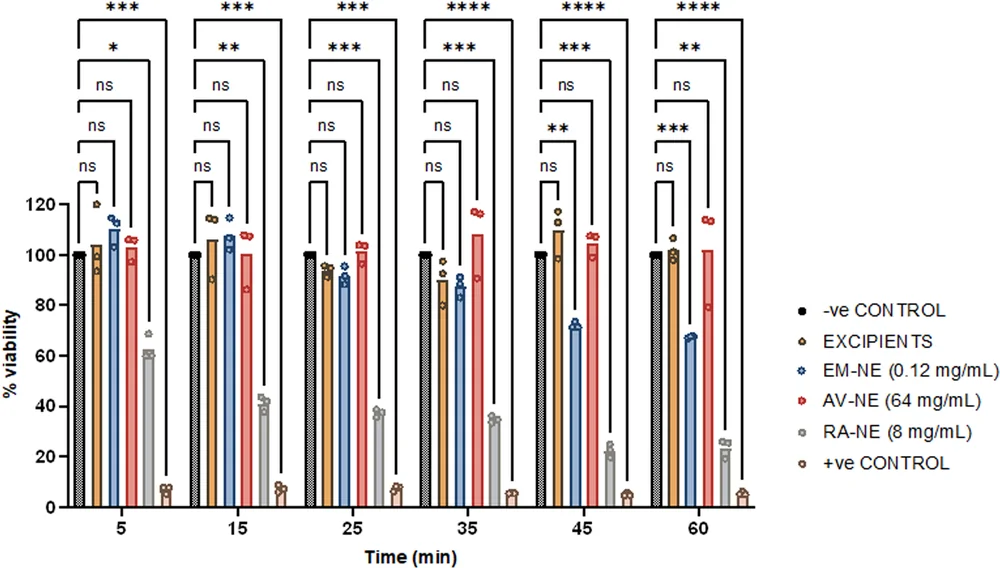
Cytotoxicity assay of nanoemulsions (NE) against human foreskin fibroblast (HFF) cells. The cells were incubated with *Artemisia vulgaris* nanoemulsion (AV‐NE) at 64 mg/mL, *Eremophila mitchellii* nanoemulsion (EM‐NE) at 0.12 mg/mL, and *Rhododendron anthopogon* nanoemulsion (RA‐NE) at 8 mg/mL. The *Y*‐axis is the percentage of viable cells compared with the negative control after 5‐ and 60‐min. Excipients (blank emulsions) represent surfactants and cosurfactants used to prepare NEs without essential oil. Negative (−ve) control represents total HFF cells in culture media, and positive control (+ve) represents HFF cells treated with 1% Triton X‐100. The error bars estimated 95% confidence interval around the mean. The results here are representative of three independent experiments. ns, nonsignificant; *significant reduction in viability *p* = 0.02; **p = 0.001; ***p < 0.0008; ****p < 0.0001 (two‐way ANOVA test followed by multiple comparison Dunnett's test). ANOVA, analysis of variance.

The safety of the EO NEs was assessed against HFF cells. No cytotoxicity was observed with the micelle mixture used to solubilize the oils at the highest concentration, with more than 90% viability after 1 h of incubation. Furthermore, the cells maintained high viability after treatment with AV‐NE at 64 mg/mL (MIC and MBEC for *S. aureus*) compared with the control group, with an average 91.43% after 1 h incubation (Figure 4). AV‐NE at 128 mg/mL (MBEC for *S. aureus* C908) also showed more than 60% active cells until 45 min, indicating cell viability amongst all NEs (Figure S3). On the other hand, EM‐NE and RA‐NE showed some toxic effects on cells, with EM‐NE toxicity being dose‐dependent. At 0.12 mg/mL (MIC value for *S. aureus* ATCC25923), approximately 70% of cells remained viable (Figure 4), while at higher concentrations of 1 and 10 mg/mL, the viability gradually declined below 60% (Figure S3). Likewise, with RA‐NE at 8 and 16 mg/mL, the viability was less than 50% after 1 h incubation (Figures 4 and S3).

## 4 Discussion

EOs from various MAPs have long been used traditionally for the treatment of various diseases or other body afflictions (Giannenas et al. 2020). Positive effects of MAPs and their EOs have been documented throughout history and were among the first pharmacological compounds used in ancient times to treat diseases and other abnormalities (Christaki et al. 2012). In modern times, approximately 3000 plants are used for their EOs, primarily in the cosmetic, food, and alternative health industries (Lubbe and Verpoorte 2011). Despite the well‐documented functional properties of EOs (e.g., antimicrobial activity), many MAPs and their EOs for medicinal purposes remain unexplored. In this study, using *S. aureus* clinical isolates recovered from CRS patients undergoing surgery as a model microorganism, we investigated the antibacterial activity of three EOs and their NEs in both planktonic and biofilm growth modes. The clinical isolates selected for this study were previously characterized and formed biofilms with varying degrees of complexity (Shaghayegh et al. 2023). The EOs used here are commercially available and mainly composed of monoterpenes and sesquiterpenes (data not published). Our results showed that all three EOs (RA‐EO, EM‐EO, and AV‐EO) had moderate antibacterial efficacy against *S. aureus*, and bacterial growth was inhibited at EO concentrations of 16, 32 and 512 mg/mL. However, their antibacterial activity was significantly improved by eightfold or more when formulated into NEs. Most importantly, the NE enhanced the antibiofilm activity of the EOs, resulting in complete eradication of biofilms in some clinical isolates at relatively low EO doses. Furthermore, the formulated NE were physically stable and biologically active for up to 6 months of storage at 4°C, indicating their long‐term stability.

EOs are mostly hydrophobic and, as such, have low solubility, cell permeability, and bioavailability, which limits their broader clinical application (Bolgen et al. 2025). EO NEs, on the other hand, have enhanced water‐solubility, cell‐permeability, and bioavailability because of their ultrafine droplet size, typically ranging between 20 and 200 nm (Jafarizadeh‐Malmiri et al. 2022). All three freshly prepared NEs in our study displayed a particle diameter below 250 nm and a negative zeta potential. Amongst all three tested NEs, the EM‐NE showed a stronger negative charge −18.3 mV compared with −2.8 mV (RA‐NE) and −0.3 mV (AV‐NE), indicating their anionic (negative) emulsion, which might explain their enhanced antibiofilm properties (Lin et al. 2021). Further, EM‐NE also had a low PDI (0.133 ± 0.01), indicating a uniform distribution of the NE droplets. As such, EM‐NE might be a candidate for antibacterial and antibiofilm applications. The differences among the NEs could be attributed to the constituents of the EOs, which also contribute to the final surface charge (Fattahi et al. 2020). It must also be noted that the negative zeta potential could partially be due to the anionic nature of (Pluronic F127), as propylene glycol is a neutral molecule, inducing slightly negative zeta potential (Saberi et al. 2013). In addition to its role as a cosolvent, propylene glycol also acts as a permeation enhancer through biological barriers (Mistry and Notman 2024), which could boost the penetration and delivery of NEs into cellular barriers and reach intracellular targets more efficiently (Nan 2015; Wulff‐Pérez et al. 2009).

**Figure 5:**
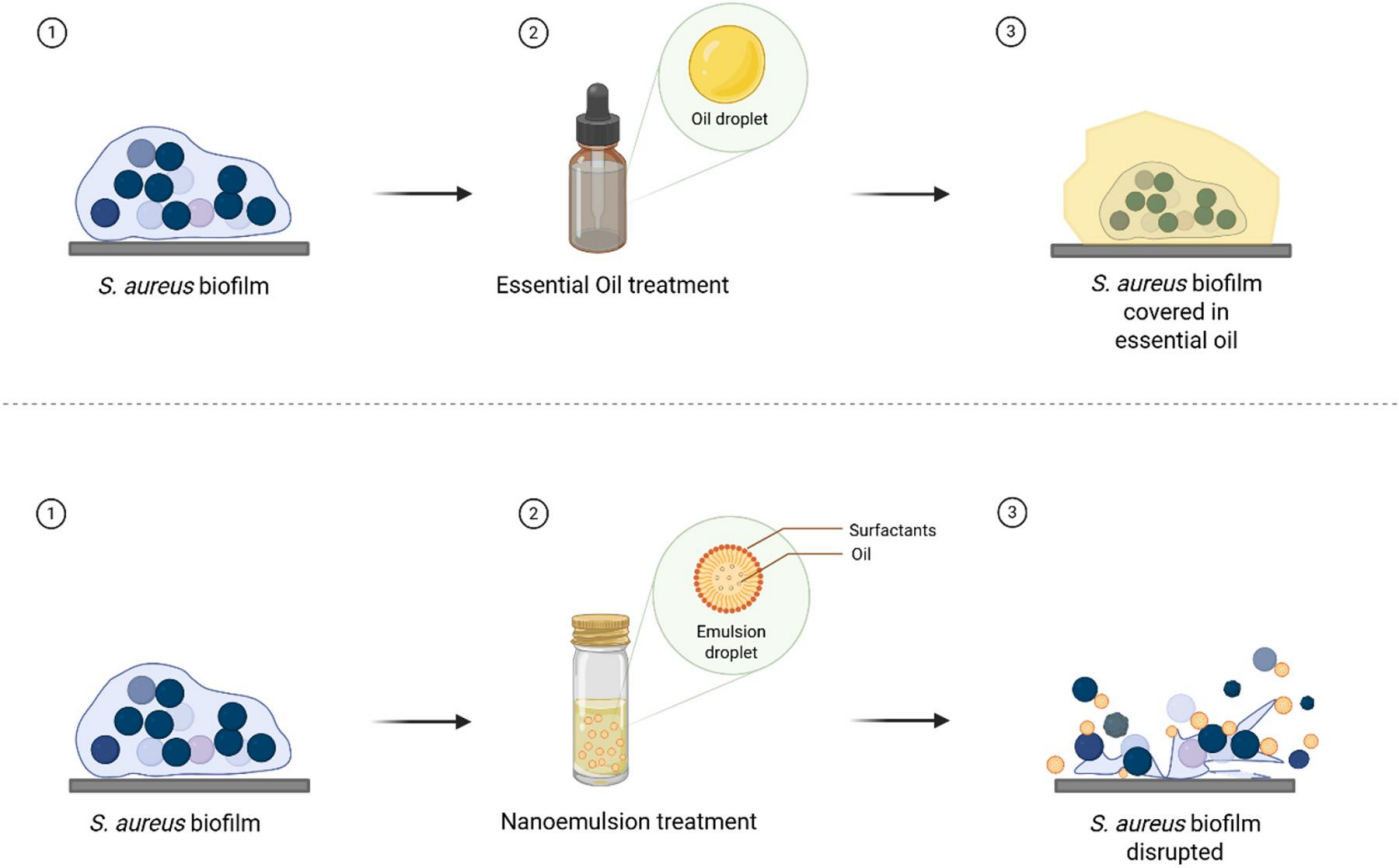
Suggested anti‐biofilm mechanism of essential oil (EO) and nanoemulsion (NE) against *Staphylococcus aureus* biofilms. The viscous EO cannot penetrate through the biofilm matrix limiting the antibacterial activity to the top layer of biofilms (upper panel) where bacteria are in direct contact with EO. However, when formulated as NE, the active ingredients of EO can diffuse into biofilms, thereby allowing stronger antibiofilm activity against bacteria located deeper within the biofilm (lower panel).

It is known that the small droplet size and high surface‐to‐volume ratio of the NEs increase surface availability of antimicrobial components in the EO for interaction with microbial cell walls, leading to membrane fusion and improved antimicrobial activity (Begum et al. 2024; Mohanta et al. 2022). Our results agree with the above theory, clearly showing that NEs inhibited the growth of *S. aureus* isolates at lower MIC and MBEC concentrations than their respective EOs, with complete elimination of bacterial biofilms at doses as low as 10 mg/mL. Similar enhanced antibacterial effects of NEs have been observed in various studies against multiple clinically important bacteria, including *Escherichia* spp., *Salmonella* spp., *Klebsiella* spp., and *Pseudomonas* spp. (Yazgan 2020; Zhang et al. 2017). Further, confocal imaging and SEM imaging showed reduced survival and inhibited adherence of *S. aureus* in the biofilms. This indicated that the active ingredients of the EO in NEs not only disrupt the cell integrity of planktonic cells but also detach biofilms, possibly by disrupting the biofilm matrix. Although the exact molecular mechanism of bacterial cell death is not yet known and may vary across different EOs, studies have suggested that the active components of NE fuse with the lipid bilayer coating of microorganisms via electrostatic attraction, driven by thermodynamic forces during the interaction (Hwang et al. 2013). This destabilizes bacterial cell membranes, disrupting their integrity and also interfering with metabolic processes, ultimately killing the microorganism (Donsì and Ferrari 2016). Furthermore, fabricating EOs into NEs form promotes the solubility and bioactivity of their active ingredients, overcoming the limitations of EO's high viscosity, which not necessarily but otherwise restricts solubility in vitro and can lead to inaccurate interpretations of antimicrobial potential (Figure 5) (Mohanta et al. 2022; Raj et al. 2022).

Because EOs are crude extracts, a single bioactive compound is rarely solely responsible for their antimicrobial properties. For example, the compound eremophilone and its oxidative derivatives are the most common amongst *Eremophila* species, but their effectiveness likely arises from the synergistic action of multiple constituents (Cock et al. 2022; Beattie 2009; Biva et al. 2016). This suggests that enhanced effectiveness of our NEs, primarily EM‐NE, may result from the increased solubility of hydrophobic bioactive compounds, allowing higher concentrations to be delivered to the target site. Importantly, all excipients used in our formulation are Food and Drug Administration (FDA) approved and considered safe (21 CFR 184.1666; 27 CFR 21.68) (Regulations 2025; CFR 2017). Our result was in alignment with the condition, as the blank micelles (excipients only in MH broth) had no antibiotic activity against standard *S. aureus* (ATCC25923). Furthermore, live/dead assays of blank micelles also confirmed no bacterial inhibition, which aligns with several other in vitro studies showing that propylene glycol and Pluronic F127 do not elicit a cytotoxic response in cells even at relatively high concentrations (Rashid et al. 2021; Abuelsamen et al. 2021). As such, formulating EOs into a more soluble form, such as NEs, using approved excipients is considered a better approach to harness bioactive natural products, as these compounds often act synergistically, resulting in enhanced antimicrobial activity compared with using the extracted active ingredients individually (Vaou et al. 2022). This opens new dimensions of research on NEs, including identifying interaction mechanisms with bacterial cells, interfering with metabolic processes, and developing products for the pharmaceutical industry, as well as creating better market opportunities for current EO producers.

Although many EOs are generally recognized as safe by the US‐FDA (Cimino et al. 2021; Angane et al. 2022), it must be noted that the toxicity of EOs largely depends on their source (plant) and may have unwanted toxic effects in addition to their antimicrobial properties. To evaluate the cytotoxic effects of NEs, we employed the MTT assay on HFF cells. The blank micelles (excipients only in DMEM media) had a good safety profile. Among the tested NEs, AV‐NE showed the highest safety profile against the tested cell line, even at a higher concentration of 128 mg/mL. While the tested concentration of EM‐NE (1 and 10 mg/mL) and RA‐NE (16 mg/mL) indicated cytotoxic effect, the cytotoxicity was concentration and time‐dependent (Figure S3), suggesting a lower concentration might be less toxic to HFF cells. As such, the protective effect of AV‐NE could be better and less toxic to cells compared with EO alone. However, it must also be noted that the physical characteristics of other EOs may adversely affect in vitro safety studies due to cross‐reactivity with the dye. For instance, *R. anthopogon* oil may have exhibited false cytotoxic activity against HFFs due to the presence of MTT enzyme inhibitors in its components, which attenuated the MTT dye signal (Ganot et al. 2013). Assessment of the safety of NEs using ex vivo and in vivo models is required to elucidate the true potential of these EOs and their NEs, given their wide application in traditional medicine. Furthermore, understanding the concentration‐dependent effects of NEs on cell viability provides crucial insights for future research and potential clinical applications.

## 5 Conclusion

Formulating EO into water‐soluble NE markedly enhanced their antibacterial and antibiofilm activity against *S. aureus*. While EOs alone showed moderate antibacterial effects, their efficacy improved severalfold when converted into NEs, with complete eradication of biofilms achieved at relatively low doses (10 mg/mL). Formulated NEs displayed small particle sizes (< 250 nm) with good physical stability and biological activity for up to 6 months, enabling efficient solubility, delivery, and stronger interactions with bacterial cells. Further, the NE maintained the viability of the tested HFF cell lines at the effective antibacterial concentrations. These results suggest that NEs overcome key limitations of EOs, such as poor solubility, high viscosity, and variability in biological activity, while preserving the synergistic action of their bioactive compounds. The core novelty of this study lies in the formulation of NEs derived from the crude EO. Although the NEs exhibited effective antibacterial properties in in vitro conditions, future studies focusing on ex vivo and in vivo are necessary to warrant the potential application of EOs NEs in pharmaceutical, dermatological, and antimicrobial applications.

## Author Contributions

**Sumeena Karki:** methodology, writing – original draft, investigation, funding acquisition. **Mahnaz Ramezanpour:** validation, supervision. **Clare M Cooksley:** project administration, resources. **Emma Barry:** project administration, resources. **Clive A Prestidge:** supervision. **Roshan Nepal:** data curation. **Sholeh Feizi:** formal analysis. **Dianne Correll:** supervision, resources. **Peter‐John Wormald:** supervision. **Sarah Vreugde:** supervision. **Muhammed Awad:** supervision, visualization, conceptualization, writing – review and editing.

## Ethics Statement

Ethics approval for the collection, storage, and use of clinical isolates and patient samples from CRS and non‐CRS control patients was granted by The Queen Elizabeth Hospital (TQEH) Human Research Ethics Committee, South Australia (HREC/15/TQEH/132).

## Conflicts of Interest

None declared.

## Supporting information


Supporting File

## Data Availability

The data that support the findings of this study are openly available in NCBI BioProject at https://www.sciencedirect.com/science/article/pii/S1521661624001128#s0170, reference number RJNA803313. The genomic sequences of the clinical isolates analyzed in this study (*S. aureus* C1033 and *S. aureus* C908) have already been published (Shaghayegh et al. 2024), and the assembled genomes are available in the NCBI BioProject database under accession code RJNA803313.
