# Supplementary material for: Nanoemulsification Promotes Antibacterial Activity of Selected Essential Oils Against Multidrug‐Resistant Staphylococcus aureus Biofilms In Vitro
Source: Microbiologyopen. 2026 Sep 9;15(5):e70407. doi: 10.1002/mbo3.70407 (PMC13559023; doi:10.1002/mbo3.70407)
Supplement: Supplementary file 1 [file MBO3-15-e70407-s001.docx]

**Figure S1: Antibiofilm activity of nano-emulsions against *Staphylococcus aureus*.** Activity of *Eremophila mitchellii* at (0.12,1 and 5 mg/mL) and *Rhododendron anthopogon* at 16 mg/mL against *S. aureus* strains. ATCC 25923 (standard laboratory strain), C1033, C908 clinical isolates from CRS patients. Data presented as mean ± SD of three biological replicate, n=3. (ns) indicate non-significant, * significant reduction in viability ** *p*= 0.0012, *** *p*<0.0001 (two-way ANOVA test followed by multiple comparison Dunnett’s test).

**SUPPLEMENTARY FIGURE**

**Figure S2: Scanning Electron Microscope image of *S. aureus* untreated (control) and treated with Essential Oil and Nano-emulsion at different concentrations**. *Eremophila mitchellii* essential oil and nanoemulsion at concentration 0.1,1,5 and 128 mg/mL were treated with *S. aureus* C1033 strain and observed under magnification 40X. EM-EO 0.1/1/5/128; *Eremophila mitchellii* essential oil at 0.1, 1, 5 and 128mg/mL concentration. EM-NE 0.1/1/5/128; *Eremophila mitchellii* nanoemulsion at 0.1, 1, 5 and 128mg/mL concentration respectively.

**Figure S3: Cytotoxicity assay of EM-NE, AV-NE and RA-NE against skin fibroblast.** MTT assay was performed on human skin fibroblast cells that were treated with *Eremophila* *mitchellii* nanoemulsion (EM-NE) at 1mg/mL and 10mg/mL, *Artemisa vulgaris* nanoemulsion (AV-NE) at 128 mg/mL and *Rhododendron anthopogon* nano-emulsion (RA-NE) at 16 mg/mL. The Y-axis is the % of fibroblast cells affected by nanoemulsions compared to the total cells treated with 1% Triton x-100 as a positive(+ve) control for 5 to 60minutes. Excipients (surfactant and co-surfactant) used to prepare nano-emulsions without EO. Negative (-ve) control represents total fibroblast cells in DMEM media. The error bars estimated 95% confidence interval around the mean. Results here are representative of three independent experiments. (ns) indicate non-significant, * significant reduction in viability *p*=0.01, ** p<0.0007, *** *p<*0.0006, *****p*<0.0001 (two-way ANOVA test followed by multiple comparison Dunnett’s test).

**SUPPLEMENTARY DATA**

**Table S1.** The antimicrobial activity of 6 months old NE of *Artemisia*, *Eremophila* and *Rhododendron* Essential Oil against *Staphylococcus aureus* ATCC 25923, and clinical isolates C1033 and C908.

| **Bacteria** | **Plant** | **Nano-emulsion containing 200 mg of EO** |
| --- | --- | --- |
|  |  | **MIC** |
| *S. aureus* ATCC 25923 | *Artemisia vulgaris* | 64 mg/mL |
| *S. aureus* C1033 | *Artemisia vulgaris* | 128 mg/mL |
| *S. aureus* C908 | *Artemisia vulgaris* | 128 mg/mL |
|  |  |  |
| *S. aureus* ATCC 25923 | *Eremophila mitchellii* | 0.125 mg/mL |
| *S. aureus* C1033 | *Eremophila mitchellii* | 2 mg/mL |
| *S. aureus* C908 | *Eremophila mitchellii* | 2 mg/mL |
|  |  |  |
| *S. aureus* ATCC 25923 | *Rhododendron anthopogon* | 8 mg/mL |
| *S. aureus* C1033 | *Rhododendron anthopogon* | 32 mg/mL |
| *S. aureus* C908 | *Rhododendron anthopogon* | 64 mg/mL |

**Table S2:** Predicted Genomic Resistance Determinants of *S. aureus* C1033 and C908 strains.

| **Isolate** | **Element symbol** | **Element name** | **Sub-type** | **Class** | **Target length** | **Reference sequence length** | **% Coverage of reference** | **% Identity to reference** | **Alignment length** | **Closest reference accession** | **Closest reference name** | **HMM accession** | **HMM description** |
| --- | --- | --- | --- | --- | --- | --- | --- | --- | --- | --- | --- | --- | --- |
| C908 | tet(38) | tetracycline efflux MFS transporter Tet(38) | AMR | TETRACYCLINE | 452 | 450 | 100 | 100 | 450 | WP_001100294.1 | tetracycline efflux MFS transporter Tet(38) | NF012176.0 | tetracycline efflux MFS transporter Tet(38) |
| C908 | ileS_V588F | Staphylococcus aureus mupirocin resistant IleS | POINT | MUPIROCIN | 917 | 917 | 100 | 99.67 | 917 | WP_000384706.1 | isoleucine--tRNA ligase IleS | NA | NA |
| C908 | blaI | penicillinase repressor BlaI | AMR | BETA-LACTAM | 126 | 126 | 100 | 94.44 | 126 | WP_001284656.1 | penicillinase repressor BlaI | NF000186.1 | penicillinase repressor BlaI |
| C908 | blaR1 | beta-lactam sensor/signal transducer BlaR1 | AMR | BETA-LACTAM | 585 | 585 | 100 | 88.55 | 585 | WP_001096386.1 | beta-lactam sensor/signal transducer BlaR1 | NF033108.1 | beta-lactam sensor/signal transducer BlaR1 |
| C908 | blaZ | penicillin-hydrolyzing class A beta-lactamase BlaZ | AMR | BETA-LACTAM | 281 | 281 | 100 | 94.66 | 281 | WP_000733281.1 | penicillin-hydrolyzing class A beta-lactamase BlaZ | NF033139.1 | penicillin-hydrolyzing class A beta-lactamase BlaZ |
| C1033 | blaI | penicillinase repressor BlaI | AMR | BETA-LACTAM | 126 | 126 | 100 | 96.03 | 126 | WP_001284656.1 | penicillinase repressor BlaI | NF000186.1 | penicillinase repressor BlaI |
| C1033 | blaR1 | beta-lactam sensor/signal transducer BlaR1 | AMR | BETA-LACTAM | 585 | 585 | 100 | 88.72 | 585 | WP_001096386.1 | beta-lactam sensor/signal transducer BlaR1 | NF033108.1 | beta-lactam sensor/signal transducer BlaR1 |
| C1033 | blaZ | penicillin-hydrolyzing class A beta-lactamase BlaZ | AMR | BETA-LACTAM | 281 | 281 | 100 | 94.66 | 281 | WP_000733281.1 | penicillin-hydrolyzing class A beta-lactamase BlaZ | NF033139.1 | penicillin-hydrolyzing class A beta-lactamase BlaZ |
| C1033 | murA_D278E | Staphylococcus aureus fosfomycin resistant MurA | POINT | FOSFOMYCIN | 421 | 421 | 100 | 99.52 | 421 | WP_000358006.1 | UDP-N-acetylglucosamine 1-carboxyvinyltransferase MurA | NA | NA |
| C1033 | murA_E291D | Staphylococcus aureus fosfomycin resistant MurA | POINT | FOSFOMYCIN | 421 | 421 | 100 | 99.52 | 421 | WP_000358006.1 | UDP-N-acetylglucosamine 1-carboxyvinyltransferase MurA | NA | NA |
| C1033 | fosB | FosB/FosD family fosfomycin resistance bacillithiol transferase | AMR | FOSFOMYCIN | 139 | 139 | 100 | 99.28 | 139 | WP_000920239.1 | FosB/FosD family fosfomycin resistance bacillithiol transferase | NF000493.1 | FosB/FosD family fosfomycin resistance bacillithiol transferase |
| C1033 | tet(38) | tetracycline efflux MFS transporter Tet(38) | AMR | TETRACYCLINE | 452 | 450 | 100 | 100 | 450 | WP_001100293.1 | tetracycline efflux MFS transporter Tet(38) | NF012176.0 | tetracycline efflux MFS transporter Tet(38) |
| C1033 | glpT_A100V | Staphylococcus aureus fosfomycin resistant GlpT | POINT | FOSFOMYCIN | 452 | 452 | 100 | 99.56 | 452 | WP_001010111.1 | glycerol-3-phosphate transporter GlpT | NA | NA |
| C1033 | glpT_V213I | Staphylococcus aureus fosfomycin resistant GlpT | POINT | FOSFOMYCIN | 452 | 452 | 100 | 99.56 | 452 | WP_001010111.1 | glycerol-3-phosphate transporter GlpT | NA | NA |
| C1033 | blaZ | penicillin-hydrolyzing class A beta-lactamase BlaZ | AMR | BETA-LACTAM | 281 | 281 | 100 | 94.66 | 281 | WP_000733281.1 | penicillin-hydrolyzing class A beta-lactamase BlaZ | NF033139.1 | penicillin-hydrolyzing class A beta-lactamase BlaZ |
| C1033 | blaR1 | beta-lactam sensor/signal transducer BlaR1 | AMR | BETA-LACTAM | 585 | 585 | 100 | 88.72 | 585 | WP_001096386.1 | beta-lactam sensor/signal transducer BlaR1 | NF033108.1 | beta-lactam sensor/signal transducer BlaR1 |
| C1033 | blaI | penicillinase repressor BlaI | AMR | BETA-LACTAM | 126 | 126 | 100 | 96.03 | 126 | WP_001284656.1 | penicillinase repressor BlaI | NF000186.1 | penicillinase repressor BlaI |

**Table S3:** Summary of antibiotic sensitivity tests against *S. aureus* ATCC 25923, C1033 and C908 strains.

| **Antibiotics** | **Strain** | **MIC (µg/mL)** | **Interpretation** | **MBEC (µg/mL)** |
| --- | --- | --- | --- | --- |
| Gentamycin | ATCC | >2 | S | >128 |
|  | C1033 | 4 | S | 128 |
|  | C908 | 8 | R | >128 |
| Ciprofloxacin | ATCC | 1 | S | >128 |
|  | C1033 | 1 | S | >128 |
|  | C908 | 2 | R | >128 |
| Clindamycin | ATCC | 1 | R | >256 |
|  | C1033 | 1 | R | >256 |
|  | C908 | 2 | R | >256 |
| Vancomycin | ATCC | ≤2 | S | >256 |
|  | C1033 | 4 | R | >256 |
|  | C908 | 4 | R | >256 |
